# DYT‐TUBB4A (DYT4 Dystonia): Clinical Anthology of 11 Cases and Systematized Review

**DOI:** 10.1002/mdc3.13452

**Published:** 2022-04-28

**Authors:** Julien F. Bally, Drew S. Kern, Conor Fearon, Sarah Camargos, Francisco Pereira da Silva‐Junior, Egberto Reis Barbosa, Laurie J. Ozelius, Patricia de Carvalho Aguiar, Anthony E. Lang

**Affiliations:** ^1^ Service of Neurology, Department of Clinical Neurosciences Lausanne University Hospital and University of Lausanne Lausanne; ^2^ The Edmond J Safra Program in Parkinson's Disease and the Morton and Gloria Shulman Movement Disorders Clinic, Toronto Western Hospital & University of Toronto Toronto Ontario; ^3^ Department of Neurology University of Colorado School of Medicine Aurora Colorado USA; ^4^ Department of Neurosurgery University of Colorado School of Medicine Aurora Colorado USA; ^5^ Department of Internal Medicine Universidade Federal de Minas Gerais Belo Horizonte Brazil; ^6^ Department of Neurology Universidade de Sao Paulo Sao Paulo Brazil; ^7^ Hospital Israelita Albert Einstein Sao Paulo Brazil; ^8^ Department of Neurology Massachusetts General Hospital Boston Massachusetts USA; ^9^ Department of Neurology and Neurosurgery Universidade Federal de Sao Paulo Sao Paulo Brazil

**Keywords:** dystonia, spasmodic dysphonia, TUBB4A, DYT4

## Abstract

**Background:**

DYT‐TUBB4A, formerly known as DYT4, has not been comprehensively described as only one large family and three individual cases have been published. We have recently described an in depth genetic and protein structural analysis of eleven additional cases from four families with four new pathogenic variants. We aim to report on the phenomenology of these cases suffering from DYT‐TUBB4A and to perform a comprehensive review of the clinical presentation and treatment responses of all DYT‐TUBB4A cases reported in the literature.

**Cases and Literature Review:**

The clinical picture was typically characterized by laryngeal dystonia (more than three quarters of all cases), associated with cervical dystonia, upper limb dystonia and frequent generalization. Extension of the dystonia to the lower limbs, creating the famous “hobby horse” gait, was present in more than 20% of cases (in only one of ours). Globus pallidus pars interna (GPi) deep brain stimulation (DBS), performed in 4 cases, led to a good improvement with greatest benefit in motoric and less benefit in laryngeal symptoms. Medical treatment was generally rather poorly effective, except some benefit from propranolol, tetrabenazine and alcohol intake.

**Conclusion:**

Laryngeal involvement is a hallmark of DYT‐TUBB4A. Symptomatic treatment with GPi‐DBS led to the greatest benefit in motoric symptoms. Nevertheless, *TUBB4A* mutations remain an exceedingly rare cause of laryngeal or other isolated dystonia and regular screening of *TUBB4A* mutations for isolated dystonias has a very low yield.

DYT‐TUBB4A,^1^ formerly known as DYT4 or “whispering dysphonia,” is an isolated dystonia first described in 1985,^2^ due to *TUBB4A* mutations. The clinical presentation of DYT‐TUBB4A has been reported in 24 patients, expressing at least one movement disorder. Patients commonly present with spasmodic dysphonia (SD) and/or a cranio‐cervical dystonia (CD) progressing to generalized dystonia. In a subsequent description of six patients from the original family (the 2 cases with concomitant Wilson disease being excluded), all six cases had both SD and CD, making combined SD and CD a frequent feature.^3^

In addition, 7 of the 24 reported patients have been reported to have a dystonic gait, in 4 of whom a distinguishing “hobby horse” gait has been emphasized.^3^ Finally, the phenotype expands further to include morphological features that cosegregated with the motor phenotype, namely a thin face and body habitus.^4^ The overall clinical presentation of TUBB4A has not been comprehensively reviewed.

In 2013, a heterozygous missense mutation, c.4C > G;p.R2G, in exon 1 of the *TUBB4A* was identified as causative in members of the original family, and another missense variant p.A271T, in a possibly familial case of segmental dystonia with SD, was identified.^4^, ^5^ In 2017 a potentially pathogenic in‐frame deletion in an Italian patient with CD was reported.^6^

In 2013, *TUBB4A* mutations were also shown to result in Hypomyelination with Atrophy of the Basal ganglia and Cerebellum (H‐ABC),^7^ or isolated hypomyelination;^8^ most of the *TUBB4A* mutations reported to date actually relate to cases suffering from H‐ABC.

In 2014 and 2015, the phenotype expanded further to include complex cases with spastic paraplegia, even more rarely reported than DYT‐TUBB4A.^9^, ^10^

In 2021, we reported four novel *TUBB4A* variants leading to DYT‐TUBB4A, all likely to be pathogenic, in eleven patients from four unrelated families.^11^ Here we provide more extensive details of their clinical histories with accompanying videos of 9 patients. Two of them underwent Globus Pallidus internus (GPi) Deep Brain Stimulation (DBS), a technique very recently reported as an effective treatment in DYT‐TUBB4A.^12^ A review of all published DYT‐TUBB4A cases to date completes this anthology, whose objective was to precisely describe the broad clinical phenotype and gather empirical information on treatment responses.

## Methods

Brazilian families were screened as part of a research project on genetics of dystonia, approved by the institutional review board of each participating center. The Canadian and American families were screened after the probands sought medical attention at our movement disorders centers. All subjects provided written informed consent.

The search for the review was conducted on PubMed, latest update 20th of July, 2021, using the words “TUBB4A,” “TUBB4,” “DYT4,” “DYT4 dystonia,” “whispering dysphonia,” “hobby horse gait.” We also browsed the congresses’ abstracts of the International Parkinson's disease and Movement Disorders Society (MDS) website. Cases of H‐ABC and spastic paraplegia, even if presenting some dystonic features, were excluded. We collected information on the clinical, imaging and therapeutic aspects of all cases included in this review.

## Results

### Case Reports

We present details on our 11 previously published cases of DYT‐TUBB4A with isolated dystonia, from four families including a singleton case, two sib pairs and a multi‐generation family. Pedigree trees and detailed genetic variants have been published previously.^11^ The detailed case reports are presented below. A video is available as a supplemental file for nine cases.

#### Family 1: Singleton, Brazilian (Brazilian Descent)

##### Proband 1.II.1

This 34 year‐old male presented with dystonia starting in the right upper limb at the age of 6. Over the next 12 years dystonia generalized involving predominantly the neck but also the trunk, upper and lower limbs and larynx, the latter resulting in marked dysphonia with a breathy whispering quality (Video 1). There were no other neurological abnormalities, except for congenital left eye divergent strabismus. He was unresponsive to levodopa, benzodiazepines, anticholinergics, propranolol or baclofen. Four brain MRI scans were normal (last one at age 30).

**Video 1:** (proband 1.II.1). Video shows a 34 year‐old male with marked cervical and right upper limb dystonia with mobile components. The dystonia extends to the cranio‐facial area, the trunk and all limbs. Spasmodic dysphonia can be heard at the end.

Family history was unremarkable for dystonia or other neurological diseases, except for a 70 year‐old uncle with Parkinson's disease (onset at 67) and a cousin with epilepsy (age not known). Examination of his parents, his brother and two paternal aunts showed no evidence of dystonia. A heterozygous SNV in *TUBB4A*, p.D295N, was found in this individual that was also present in unaffected family members (father 1.I.1, sibling 1.II.2 and two paternal aunts 1.I.2 and 1.I.3); in silico analysis suggested that it is deleterious.^11^ A different rare variant at the same residue, p.D295H, is described in gnomAD^13^ in a single individual of European descent (allele frequency of 8.8 x 10^−6^).

#### Family 2: Sib Pair, Brazilian (Portuguese Descent)

##### Proband 2.II.1

This 50 year‐old woman developed sudden onset of right hand finger flexion at the age of 30 and also a right upper limb inversion when walking, leading especially to writing disability. Shortly afterwards, her voice became strangled with some breaks. Levodopa‐carbidopa led to some improvement in voice quality. She also described a slight improvement with alcohol. Three years after onset she developed a left torticollis responsive to botulinum toxin. Recent neurological exam (Video 2) demonstrated generalized dystonia with objective Burke‐Fahn‐Marsden (BFM) scale movement score of 24 points, plus 10 points in disability. Brain MRI and laboratory work up did not demonstrate any abnormalities.

**Video 2:** (proband 2.II.1). Video shows the woman when she was 44 years old, with a cranio‐facial and cervical dystonia (with occasional dystonic tremor) and milder involvement of the trunk and all 4 limbs. There is a marked writer's cramp. The voice is slightly strained.

##### Case 2.II.2 (Brother)

This 44 year‐old man developed a strangled voice (spasmodic dysphonia (SD)—adductor type) and dysphagia at the age of 21. One year later he developed a dystonic posture of the right upper limb (internal rotation of the arm, forearm pronation, flexion of the wrist), trunk dystonia, left laterocollis and valgus knees with internal rotation of the lower limbs. Objective BFM scale movement score was 66, plus 19 points in disability. MRI and laboratory work up (including ceruloplasmin and copper) did not demonstrate any abnormalities. Treatment with levodopa‐carbidopa, biperiden, trihexyphenidyl and clonazepam failed to improve either dystonia or pain, and nine years after the onset of symptoms, the patient underwent a left pallidotomy with improvement of the right dystonia (2003). With progression of dystonia on the left side, a right palidotomy was performed with a slight but significant improvement in anarthria and dysphagia. Objective BFM scale score after surgery was 52 (movement) plus 13 (disability). The patient was lost to follow up in 2008 but according to his sister he remains stable.

A heterozygous *TUBB4A* variant, p.R46M, was present in both affected members. There was no other relevant family history (the deceased father had developed what may have been right foot dystonia in his forties following a stroke).

#### Family 3: Multigenerational, Six Definitely Affected Members, Canadian (French Descent)

##### Proband 3.III.6

This woman developed spasmodic dysphonia at age of 10. Over the next several years she developed severe generalized dystonia. Despite multiple drug trials and a right thalamotomy at age 14, bilateral C1‐3 rhizotomy at age 17 for severe retrocollis and left thalamotomy at age 19, she remained severely disabled and chair‐bed bound with generalized dystonia particularly involving speech and phonation, axial muscles (severe retrocollis and axial extension) and right arm (Video 3). Pain was also a prominent feature. For a time she did seem to benefit from a combination of trihexyphenidyl, pimozide and diazepam (Video 3— second segment). At age 37 she underwent bilateral GPi DBS with a 45% improvement of the BFM scale movement subscore (78 to 43) and a 36% improvement of the BFM disability subscore (22 to 14); Toronto Western Spasmodic Torticollis Rating Scale (TWSTRS) severity subscore improved by 17% (18 to 15) and pain completely resolved (13.75 to 0); pain was therefore most probably due to the dystonia. Unfortunately, the patient died 6 months after surgery from a fall from a balcony. The family denied the possibility of a suicide. MRI scan performed prior to DBS was unremarkable.

**Video 3:** (proband 3.III.6). First segment shows the female when she was 19 years old. Note the severe spasmodic dysphonia, the neck extension and arching of the back and the right arm dystonia. She was chair‐bed bound. Second and third segments show her when she was 24 years old, when she transiently responded to oral drugs and could walk. However the laryngeal dystonia and back arching did not improve.

##### Case 3.III.5 (Brother)

This 54 year old man was well until age 21 when he described “mumbling” of speech; at the age of 23 he developed a strangled voice characteristic of adductor SD. Writer's cramp (WC) affecting his right arm developed over the next two years (Video 4). He also had long‐standing motor and phonic tics indicative of a diagnosis of Tourette syndrome; his father and paternal uncle, who had no features of dystonia, had tics since childhood. Trihexyphenidyl initially had a striking effect on SD but no impact on WC. Later he received botulinum toxin injections for the spasmodic dysphonia. He was last seen by the authors at age 36. At that time he was more bothered by his tics than the very evident SD and WC (Video 4, third segment). Examination at that time also showed questionable dystonia in the left‐hand and mild bilateral foot dystonia. MRI scan was not obtained.

**Video 4:** (case 3.III.5). First segment shows the man when he was 26 years old, with marked spasmodic dysphonia responding to shouting and singing. Second segment shows him a year later with laryngeal dystonia much better on trihexyphenidyl. Third segment, when he was 35 years old, however shows marked laryngeal dystonia again, without treatment. Last segment shows a writer's cramp with right thumb adduction and extension of the distal phalange when he draws circles with his non‐dominant left hand.

##### Case 3.II.3 (Mother)

This 45 year‐old (when first seen) woman developed right writer's cramp in her early 20s. At the same time she noted a mild intermittent bilateral action tremor which had changed little over the years. At the age of 18 she noted a brief (5–10 minutes) episode of side‐to‐side headshaking at a time of stress. Shortly before she was first seen she began experiencing daily, very brief episodes of involuntary head turning to the right (with 2 occasions to the left) associated with pain and cracking in the back of the neck which would subside immediately after the head deviated. Examination revealed only dystonia on writing with the right hand without evidence of dystonia in the neck or other body regions (Video 5). She remained clinically unchanged over the following 18 years. MRI was not obtained.

**Video 5:** (case 3.II.3). First segment shows the physician describing the patient's complaints, as only few signs were visible on clinical exam. Second segment shows a writer's cramp, with progressive flexion of the fingers at the proximal phalangeal joint and extension at the distal phalangeal joint. Her compensatory posture consists in holding the pen without using the fingers’ pulp.

##### Case 3.II.1 (Maternal Aunt)

This 57 year‐old woman underwent surgery on her right shoulder for an injury at the age of 25. Shortly after this she had a “nervous breakdown” and about the same time experienced severe spasms in her right arm followed by sudden jerking of her head backwards and to the right side. Subsequently she developed blepharospasm (at times she was functionally blind), and severe jaw closing dystonia causing tooth damage which she could overcome by inserting her finger between her teeth. Later she developed involvement of the left arm and legs. Between the ages of 32 and 33 (in the early 1960s) she underwent five stereotactic brain operations (most likely involving the thalamus) as well as surgery on her forehead muscles to relieve blepharospam. The last stereotactic procedure involving the left hemisphere was followed by a “stroke” with persistent right hemiplegia. She had been treated with haloperidol with some benefit but then developed a parkinsonian rest tremor in the left arm which varied depending on dosage. At the time of her only assessment in 1987 (Video 6) she demonstrated a slightly strained voice without vocal breaks, craniofacial dystonia, CD (particularly with retrocollis), bilateral upper and lower limb dystonia. Of note, in addition to dystonia, the clinical picture included right hemiparesis and spasticity (due to the stroke) and a parkinsonian resting tremor in the left arm (presumably related to her haloperidol therapy). MRI was not obtained.

**Video 6:** (case 3.II.1). First segment shows the patient and the physician describing the complaints. One can hear a slightly strained voice and see a retrocollis with neck dystonic tremor and cranio‐facial dystonia. The patient describes her “geste antagoniste” to relieve the jaw closing dystonia. On exam spasticity is evident on the right side, whereas slight dystonia is present on the left, along with a drug‐induced parkinsonian syndrome.

##### Case 3.III.2 (Maternal Cousin)

This woman was seen on two occasions in her mid‐30s in the 1980s (records no longer available). She developed dystonia involving her voice, face, neck and upper limbs at age 24. Like her mother (case 3.II.1), the dystonia partially responded to dopamine receptor blockers. On examination (Video 7), like her mother, her voice was mildly strained but easily understood without vocal breaks. She had dystonia involving the face and neck (especially with anterior/forward displacement but occasionally demonstrating retrocollis) and bilateral upper limbs. She did not complain of lower limb involvement but there was mild dystonic posturing in both feet. MRI was not performed.

**Video 7:** (case 3.III.2). First segment shows evident cranio‐facial, neck (mainly flexion and anterior sagittal shift) and both upper limbs dystonia. The voice is almost normal. Trunk dystonia is evident on standing. Second segment shows her 5 years later with worsening of the laryngeal dystonia but some improvement in the cervical dystonia. Feet were only very slightly involved.

##### Case 3.III.4 (Maternal Cousin)

This patient developed dystonia involving the neck at age 7 followed by the left foot. Severe generalized dystonia rapidly progressed, leading to death at age 13. She was not genetically tested but was definitely clinically affected, hence the inclusion to Table 1.

**Table 1:** Clinical features of all reported DYT‐TUBB4A cases to date (only isolated dystonia; H‐ABC syndrome excluded); *duplicate cases are in italic*

First author	Year of publication	Number of affected cases reported	Age at onset (in bracket: age used to calculate SD)	Spasmodic dysphonia (SD)	Cervical dystonia (CD)	Generalized dystonia (= trunk + minimum 2 segments)	Dystonic gait	Other body sites of dystonia	Other neurological symptoms	Died at age	Treatment	Commentary
**Parker N.**	**1985**	**N = 14 with at least one movement disorder** (total N = 18 plus 7 suspect cases)										
		Case I‐B	50s (50)	‐	‐	‐	Yes			‐	‐	
		^a^Case II‐F		Yes, severe	‐	‐	‐	Champing movements of the jaw; hypertonus all limbs (likely reflecting dystonia)	Demented	‐	‐	
		Case II‐M	30s (30)	‐	‐	‐	‐	Choreiform movements^c^		46	‐	
		^a^Case III‐L	‐	‐	‐	‐	‐	Choreiform movements^c^	Demented	‐	Haloperidol; electro‐shock treatment: improvement	
		Case III‐R	30s (30)	‐	‐	‐	‐	Choreiform movements^c^		‐	‐	
		Case III‐T	20s (20)	Yes	‐	‐	‐	Choreiform movements^c^	Demented; aggressive; suicidal attempts	‐	‐	
		Case III‐V	30s (30)	‐	‐	‐	‐	Choreiform movements^c^	Dysphagia; breathing difficulties	32	‐	
		Case III‐W	Late 30s (35)	Yes, mute (except when shouting)	Yes	Yes	‐	Blepharospasm	Kyphoscoliosis	‐	‐	
		^a^Case IV‐A	22	Yes, severe (present at onset)	‐	‐	‐		Dysphagia	‐	Crico‐pharyngeal myomectomy: marked but transient improvement	
		Case IV‐P	‐	Yes (isolated & present at onset)	‐	‐	‐			‐	‐	
		^a^Case IV‐S	< 19 (18)	Yes, marked (present at onset)	Yes, intermittent	‐	‐		IQ of 68; inadequate behavior	‐	‐	
		Case IV‐W	‐	Yes, mild (isolated & present at onset)	‐	‐	‐			‐	‐	
		^a^Case IV‐X	23	Yes (present at onset)	Yes	Yes	‐			‐	Stereotactic lesions in thalamus: marked but transient improvement; Anti‐parkinsonian drugs: no improvement	
		Case IV‐AB	‐	Yes, mild (isolated & present at onset)	‐	‐	‐			‐	‐	
**Wilcox R.**	**2011**	**N = 5 newly reported cases** *(N = 8)*									‐	**Same family as Parker N**.
		^a^ *Case V‐1*	*22*	*Yes, severe*	‐	*Yes*	*Yes, moderate hobby horse gait*			‐	*Alcohol response: yes*	*Same as case IV‐A from Parker*
		^a^Case V‐3	24	Yes, severe	‐	Yes	Yes, moderate hobby horse gait			‐	Good response to propranolol 40 mg BID	
		^a^ *Case V‐7*	*19*	*Yes, moderate*	‐	*Yes*	*Yes, hobby horse gait*			‐	*Bilateral pallidotomy: probably no improvement; propranolol: no response; alcohol response: yes*	*Same as case IV‐W from Parker*
		^a^ *Case V‐8*	*23*	*Yes, severe*	‐	*Yes*	‐		*Upper limb tremor (unclear whether this was a dystonic tremor)*	‐	*Bilateral ventrolateral nuclei thalamotomy: probably no improvement; mildly propranolol (10 mg BID) responsive; alcohol response: yes*	*Same as case IV‐X from Parker*
		^a^Case V‐9	42	Yes, moderate	‐	‐	‐			‐	No propranolol response at 40 mg BID; alcohol response: yes	
		^a^Case V‐17	17	Yes, moderate	‐	‐	Yes			‐	Useful propranol response at 40 mg BID; response to botulinum toxin for SD; alcohol response: yes	
		^a^Case VI‐5	29	Yes, moderate	‐	‐	Yes, very mild			‐	No significant response to propranolol at 40 mg BID; response to botulinum toxin; alcohol response: yes but mild	
		^a^Case VI‐11	17	Yes, severe	‐	Yes	Yes, hobby horse gait			‐	Robust propranol (80 mg BID) and tetrabenazine (25 mg BID) response with improvement of tongue extrusional dystonia and hobby horse gait; good initial response to botulinum toxin for SD; alcohol response: yes	
**Lohmann K.**	**2013**	**N = 1 newly reported cases** *(N = 2)*										**Genetic study screening the same family as Parker N. and Wilcox R.**
		^*a*^ *Index case L‐3270*	*20*	*Yes, severe*	*Yes*	*Yes*	*Yes, hobby horse gait*	*Tongue protrusion (likely dystonic)*	*Severe dysphagia; eyelid ptosis; characteristic facies and body habitus*	‐	*Good relief of dystonic symptoms with alcohol*	*Same as case VI‐11 from Wilcox*
		Unrelated case, after screening of 394 unrelated dystonia patients	60	yes	‐	‐	‐	Oromandibular dystonia and dyskinesia		‐	‐	
**Hersheson J.**	**2013**	**N = 2 newly reported cases** *(N = 6)*										**Genetic study screening the same family as Parker N. and Wilcox R.**
		*Case V‐2*	*21*	*Yes*	*Yes*	‐	*Yes*		*Swallowing difficulties*	‐	‐	*Possibly same as case IV‐A from Parker*
		Case V‐14	37	Yes	Yes	Yes	‐		Swallowing difficulties	‐	‐	
		Case V‐16	30	Yes	Yes	‐	‐			‐	‐	
		*Case V‐18*	*13*	*Yes*	*Yes*	‐	‐			‐	‐	*Possibly same as case IV‐W from Parker*
		*Case V‐20*	*28*	*Yes*	*Yes*	‐	‐	*Left hemidystonia*		‐	‐	*Possibly same as case IV‐X from Parker*
		*Case V‐24*	*23*	*Yes*	*Yes*	‐	‐	*Tongue and limb dystonia*		‐	‐	*Possibly same as case IV‐AB from Parker*
**Airey C.F.**	**2013**	**N = 1**										**Poster at MDS congress**
		*Single case*	‐	‐	‐	*Yes*	*Wheelchair dependance*	*Extrusional tongue dystonia*	Speech and swallowing difficulties	‐	*GPi‐DBS: improvement on tongue extrusional dystonia, speech, swallowing and walking capacity*	*Confirmed as same as case VI‐11 from Wilcox and index case from Lohmann*
**Xia M.‐A.**	**2015**	**N = 1**										**Abstract**
		*Single case*	‐	*Most probably*	‐	*Yes*	*Skipping gait*	*Extrusional tongue tremor (likely dystonic)*	*Mute*		*Ventral posterolateral nucleus [of the thalamus] DBS: significant improvements on the swallowing skills, walking capacities, speaking skills and general dystonia*	*Probably from the same family described by Wilcox* et al.
**Vulinovic F.**	**2017**	**N = 1**										
		Single case	21	‐	Yes	‐	‐			‐	‐	Lost to follow‐up at age 35
**Delorme C.**	**2021**	**N = 1**										
		Single case	25	Yes	Yes	Yes	‐	Facial involvement	Mild pyramidal and cerebellar features	‐	Anticholinergics and botulinum toxin: poor effect; GPi‐DBS resulted in a 55% reduction of dystonia (facial and cervical areas)	
**Bally J.F.**	**2021**	**N = 11**										**Report of 4 distinct unrelated families**
		^a^, ^,b^ Proband 1.II.1	6	Yes	Yes	Yes	No	Right upper limb		alive	Unresponsive to levodopa, benzodiazepines, anticholinergics, propranolol or baclofen	
		^a^, ^**,b**^ Proband 2.II.1	30	Yes	Yes	Yes	No	Upper limbs		alive	Levodopa‐carbidopa: some improvement in SD; slight improvement with alcohol; CD responsive to botulinum toxin	
		^a^ Case 2.II.2	21	Yes	Yes	Yes	No	Upper & lower limbs		alive	Levodopa‐carbidopa, biperiden, trihexyphenidyl and clonazepam: no improvement; staged bilateral pallidotomy: improvement	
		^a^, ^**,b**^ Case 3.II.1	25	Yes	Yes	Yes	No	Cranio‐facial & right upper limb		LFU	Five stereotactic brain operations: no clear improvement; haloperidol: some improvement	
		^a^, ^**,b**^ Case 3.II.3	18	No	Yes	No	No	Right upper limb		LFU		
		^a^, ^**,b**^ Case 3.III.2	24	Yes	Yes	Yes	No	Cranio‐facial & upper limbs		LFU	Dopamine receptor blockers: some improvement	
		Case 3.III.4	7	Yes	Yes	Yes	No			13		Very severe
		^a^ ^,^ ^b^ Case 3.III.5	21	Yes	No	No	No	Right upper limb		LFU	Trihexyphenidyl: striking but transient effect on SD; botulinum toxin for SD	
		^a^ ^,^ ^b^ Proband 3.III.6	10	Yes	Yes	Yes	No	Cranio‐facial, upper & lower limbs		37	Staged bilateral thalamotomy: no improvement; combination of trihexyphenidyl, pimozide and diazepam; slight improvement; bilateral GPi‐DBS: 17 to 43% improvement on different scales (BFM and TWSTRS) & pain completely resolved	
		^a^ ^,^ ^b^ Proband 4.II.2	2	Yes	Yes	Yes	Yes, hobby horse gait	Cranio‐facial, upper & lower limbs		alive	Botulinum toxin for SD: no improvement; carbidopa/levodopa, baclofen, benzodiazepines, tetrabenazine: no improvement; Marked improvement after GPi DBS	
		^a^ ^,^ ^b^ Case 4.II.3	22	No	Yes	Yes	No	Upper limbs		alive	Left cervical rhizotomy: marked improvement of CD	
**TOTAL & Percentage over total cases (denominator = 35)**		**N = 35**	**Mean age at onset: 24.9y; Standard deviation: 12.0y; Median: 23.5y; Range: [2‐60y]**	**27 (77%)**	**21 (60%)**	**17 (49%)**	**8 (23%)**	**Limb involvement reported: 14 (40%); Cranio‐facial and/or oro‐pharyngeal involvement reported: 14 (40%)**			**‐Alcohol response: 7** **‐Propranolol response: 4** **‐Tetrabenazine response: 1** **‐Botulinum toxine response: 5 ‐Stereotactic lesional procedures (thalamus, pallidum): unclear response; ‐GPi‐DBS response: cases good improvement in 4 cases over 4 procedures** **‐ventral posterolateral DBS: good improvement in 1 case over 1 procedure**	
^d^Percentage over documented signs (numerator/denominator)				93% (27/29)	95% (21/22)	89% (17/19)	44% (8/18)					

SD: spasmodic dysphonia; CD: cervical dystonia; y: years; −: not documented; LFU: lost to follow‐up.

‐in the Parker 1985 series, only suspected cases including at least one movement disorder are mentioned.

‐the 2 Parker cases suffering from Wilson's disease (IV‐AD in Parker = V‐27 in Hersheson; IV‐AE in Parker = V‐26 in Hersheson) are not reported, but one of them suffered from severe dysarthria and the other from severe dystonic gait.

‐Case V‐5 from Wilcox series is not included because he was adopted out of the family.

*‐Duplicate/triplicate cases are in italic; a given symptom might be mentioned only in one or two of the reports*.

Dysphagia and swallowing difficulties have been placed under “other neurological symptoms” but these could also have easily been due to dystonia.

^a^ examined by the first author (in Parker and Wilcox articles) or a co‐author.

^b^ video available as a supplemental file.

^c^ “choreiform movements” described by Parker likely reflect rapid phasic dystonia.

^d^ percentage of presence of signs over total documented presence or absence of signs.

The following 3 cases are not included in Table‐1, as genetic testing was either not done (Cases 3.I.1 and 3.II.2) or revealed wild‐type (case 3.III.3).

##### Case 3.III.3 (Maternal Cousin)

This lady was seen once in her mid‐30s only as part of screening for relatives of the individuals in her family known to be affected by dystonia. She had no neurological complaints (records no longer available). On examination there was reduced right arm swing, very questionable right‐hand dystonic posture and possibly some tightness on writing with the right hand (claimed to have always written this way) but no mirror dystonia on writing with the left. Her voice was normal.

##### Case 3.I.1 (Maternal Grand‐Mother)

This deceased woman was reported as suffering from “bad tremor” which, according to the description from her daughter, was consistent with either “essential” tremor or dystonic tremor.

##### Case 3.II.2 (Maternal Uncle)

This man was seen once in his mid‐50s. He had a 15 year history of tremor in the head and hands. Since childhood he had had tension and pulling of the head to the right at variable speeds, typically associated with urge and relief following the head movement. He also complained of long‐standing excessive blinking. On examination there was only a tremor in the right‐hand seen while writing, holding a posture and less with action.

#### Family 4: Two Definitely Affected Members, USA (Norwegian and Czech Descent)

##### Proband 4. II. 2

This is a 64 year‐old right handed woman with onset of hypophonia at 2 years of age. At 8 years of age, she developed gait impairment characterized by difficulty with hip flexion. Her symptoms minimally progressed until 53 years of age. Over three years, her speech progressively worsened becoming unintelligible and her gait became more impaired necessitating the use of a walker. She developed panic attacks at 59 years of age. Over the next four years she required assistance for independent activities of daily living. She was unable to stand without the use of a walker and preferred to crawl to mobilize around her home. She used sign language and a voice amplifier to communicate. Previous treatment with botulinum toxin injection into her vocal cords and medication trials including carbidopa/levodopa, baclofen, benzodiazepines, tetrabenazine as well as biofeedback were ineffective. On examination, she had profound spasmodic dysphonia and generalized dystonia. She had abnormal posturing with radial forearm deviation and internal rotation of her left foot, right shoulder elevation and right laterocollis. She was unable to fully open her jaw, protrude her tongue and had limitation of neck mobility. Mobile dystonia was greatest on the left side of the body and she had blepharospasm. With handwriting, she assumed a posture with knees flexed and arms partially extended. Gait was characterized by bilateral knee flexion, right hip flexion and left hip extension (“hobby horse gait”) (Video 8). TWSTRS score was 26; BFM movement score was 89, plus 20 in disability. Neuropsychological evaluation was normal. Brain MRI revealed mild diffuse global atrophy, mild scattered deep and subcortical cerebral white matter disease.

**Video 8:** (proband 4.II.2): pre‐DBS and 7 months post‐DBS. Baseline and 7 months post‐operative bilateral GPi DBS. At baseline, patient had severe spasmodic dysphonia, marked cervical dystonia with laterocollis and right shoulder elevation, cranio‐facial dystonia and upper limb bilateral mobile dystonia with dystonic tremor on the left. Gait without a walking device is only possible by crawling. With the help of a walker she manages to walk: marked lower back extension and bilateral lower limb dystonia are evident, creating a kind of “hobby horse gait.” At 7 months post bilateral GPi DBS implantation demonstrating marked improvement in all symptoms with near complete resolution of mobile dystonia and dystonic tremor.

The patient underwent bilateral GPi non‐staged DBS implantion with Medtronic 3389 DBS leads and Medtronic Percept PC implanted pulse generator.

Post‐operative at seven months, she no longer used assisted devices to speak and many spoken words were able to be understood. Abnormal posturing was remarkably improved along with mobile dystonia and she had almost complete resolution of blepharospasm. Handwriting was easier to perform with improved legibility. She was able to walk independently but preferred to use a walker due to impaired balance. TWSTRS score was 17; BFM movement score was 34.5 (respectively 35 and 61% improvement in the dystonia rating scales) ◊ (Post‐DBS Video 8).

##### Case 4. II. 3 (Brother)

This is a 61 year‐old man with onset of left arm irregular tremor occurring with action at age of 22 years. Tremor progressed to involve the right arm and he developed torticollis at 26 years of age. He underwent left cervical rhizotomy at 27 years of age with reported marked improvement of abnormal head posturing. At this age, he also had a spinal cord stimulator implanted but experienced “shock like” sensation of his neck and subsequently turned it off shortly after implantation. At 44 years of age, he underwent cervical spinal fusion. Over the past 15 years, the patient reports that cervical dystonia has remained stable but endorses that arm tremor gradually has worsened. On examination, he had slight mobile dystonia greatest with arms extended in supination and in winged posture. He had elevation of the right shoulder and limited neck mobility. Patient had scoliosis and he was slightly stooped (Video 9). TWSTRS score was 18; BFM movement score was 18.

**Video 9:** (case 4.II.3). Video shows marked cervical dystonia with diminished range of motion (cervical spinal fusion), bilateral upper limb dystonia more evident on the right. Gait, which is of good quality, is characterized by a stooped posture and lateral deviation of the trunk to the left.

### Literature Review

The PubMed search retrieved the following results: “TUBB4A” (yielding 86 articles) “TUBB4” (yielding 28 articles), “DYT4” (yielding 27 articles), “DYT4 dystonia” (yielding 26 articles), “whispering dysphonia” (yielding 25 articles), “hobby horse gait” (yielding 3 articles). Only 7 of these articles, including ours, described cases with mutations in the *TUBB4A* gene and a phenotype consistent with dystonia,^2^, ^3^, ^4^, ^5^, ^6^, ^11^, ^12^ after exclusion of articles reporting on H‐ABC and spastic paraplegia. However 4 of these articles describe the same original family from Parker, adding new cases from the same family^2^, ^3^, ^4^, ^5^; in 1 of these 4 articles, a case from another family is also reported.^4^ The 2 remaining articles report single cases.^6^, ^12^ In total, there are 4 isolated cases, two sib pairs and 2 large families reported to date. Including only the cases with sufficient clinical descriptions, this leads to a total of 35 cases of DYT‐TUBB4A to date (Table 1).

Searching the MDS website for Congresses abstracts retrieved one poster^13^ and one abstract,^14^ from the same Australian group, describing DBS performed in DYT‐TUBB4A: the poster reports improvement on the patient's tongue extrusional dystonia, speech, swallowing and walking capacity, after GPi‐DBS. The patient is the same as the VI‐11 case from Wilcox et al.^3^ and the index patient from Lohmann et al.^4^ (information confirmed by the poster's first author). The abstract reports significant improvements after ventral posterolateral thalamic nucleus DBS on swallowing, walking capacities, speaking and general dystonia, permitting withdrawal of all her dystonia medications. This patient probably also belongs to the same original Australian family.

Table 1 summarizes the clinical features and response to treatment of the 35 cases reported in the 7 articles, the poster and the abstract. SD was reported in 77% of cases (27/35), CD in 60% (21/35), generalization of the dystonia in 49% (17/35) and dystonic gait in 23% (8/35). Cranio‐facial and/or oro‐pharyngeal involvement of the dystonia was reported in 40% of cases (14/35). Limb involvement was reported in 40% of cases (14/35), being bilateral in 6 out of the 14 cases (43%), unilateral in 5/14 (36%) and not stated in 3/14 (21%). Percentages of presence of a given sign over total documented presence or absence of this sign represent 93% (27/29) for SD, 95% (21/22) for CD, 89% (17/19) for generalized involvement and 44% (8/18) for dystonic gait.

Cranial MRI scans of the affected individuals were generally unremarkable.^3^, ^11^

## Discussion

Herein, we provide in‐depth descriptions of 11 definitely affected cases, including 2 with response to GPi‐DBS, and have reviewed an additional 24 cases from the literature. Age at onset has varied considerably from early infancy to late adulthood. Interestingly, age at onset was not predictive of outcome, as 1 case developing in childhood died as a consequence 6 years later, while another case developing dystonia as a toddler is still alive at 64 years old, and markedly benefitted from DBS at this age. This latter case interestingly had a late‐onset severe worsening after a long period of stability, a course that is somewhat unusual in other forms of genetic isolated dystonia.

The most consistent feature of DYT‐TUBB4A is laryngeal involvement, present in more than 3 quarters of reported cases, making it a hallmark feature. The opposite is not true, as screening for *TUBB4A* mutations in isolated SD gives extremely low yield.^15^, ^16^ Other isolated dystonia genes associated with laryngeal dystonia include *TOR1A* (DYT1), *THAP1* (DYT6) and *GNAL* (DYT25).^17^ In the original report, Parker very precisely described the type of SD present in DYT‐TUBB4A patients and the pleiotropic clinical expression: “*They are able to shout and yell when emotional, have no trouble communicating after drinking alcohol and talk normally in their sleep, yet when they try to speak their voices come out only in a faint whisper. Eventually they may be unable to utter a sound when trying to talk. This whispering dysphonia may continue throughout life as an isolated feature, but more commonly is the initial presentation of a more pervasive disease with extremely varied expression*.” This description of the SD in DYT‐TUBB4A concurs with our experience (e.g., Video 4). Interestingly, GPi‐DBS improved SD in proband 4.II.2 (see Video 8 post‐DBS).

Other consistent features included CD, present in 60% of the reported cases, upper limb dystonia and frequent generalization of the dystonia in about half of the cases. Although it was present in only one of our cases, the “*hobby horse gait*,” highlighted in previous papers, was found in close to a quarter of cases, with remarkable improvement after GPi‐DBS in our patient (Video 8 post‐DBS) and after propranolol and tetrabenazine in case VI‐11 from Wilcox.

In the Wilcox series, no diurnal variation in dystonic symptoms and no ameliorating geste antagoniste were reported. None of our patients spontaneously reported diurnal fluctuations; at least one (3.II.1) did note benefit with sensory tricks for blepharospasm and jaw closing dystonia. In the Lohmann series, morphological features that completely cosegregated with the motor phenotype were highlighted, namely a thin face and body habitus; this was evident in at least 2 of our cases (3.III.6 and 3.III.2). It is not clear whether this is a feature of the disorder or simply due to swallowing difficulties or increased energy expenditure in persons with more severe involuntary movements.

DYT‐TUBB4A cases have originated from a variety of ethnic backgrounds (English, French, Italian, Portuguese, Norwegian, Czech), although to date they have all been of European origin. Penetrance has been quite variable in available pedigrees. In some previously reported families it seems to be rather high; for example, in the Wilcox series, one affected woman had seven children, 4 definitely affected and 2 suspected; another woman had four out of five affected children. However, in our series, some variants were also present in unaffected family members, probably due to reduced penetrance.

Brain imaging in DYT‐TUBB4A, unlike in H‐ABC syndrome, is usually unremarkable.^18^

Regarding medical treatment, most drugs tried had a rather poor effect, or only led to partial improvement. An important exception relates to 1 article reporting significant improvement after either propranolol (n = 4), tetrabenazine (n = 1) or alcohol intake (n = 7).^5^ A video provided along with the article shows one case (VI‐11, same as Lohmann's index case) with a very marked improvement of the tongue protrusion dystonia and the hobby horse gait on a combination of propranolol and tetrabenazine; it is not clear which of these contributed most to this improvement. Of note, this patient is the same one who responded to the GPi‐DBS, presented as a poster (see below and above); the DBS improvement was reported in 2013, whereas the drug improvement was reported in 2011. Botulinum toxin injections, either to treat SD or CD, were also effective in some cases (n = 5).

GPi‐DBS proved effective in 2 of our cases (including providing benefit for SD in one) as well as in 1 case reported in the literature,^8^ and a second case reported as a poster.^13^ Ventral posterolateral nucleus [of the thalamus] DBS was also reported once, with a good outcome.^14^ Stereotactic lesional procedures were performed in the thalamus in two cases (one with no improvement, the other one with only transient benefit), in the pallidum in two cases (one with improvement, one without). Another case underwent five stereotactic lesional procedures (no precise brain region mentioned but knowing the center these most likely involved the thalamus) without any sustained benefit. Left cervical rhizotomy led to a marked improvement of CD in one case and crico‐pharyngeal myomectomy to a marked but transient improvement in another case.

### Limitations

Lack of information does not equal negative information; it is possible that more of the Parker and Wilcox cases also suffered from CD, a syndrome infrequentely reported in these series, thus decreasing the total percentage of cervical involvement in DYT‐TUBB4A. Further, Parker mentioned that “*others have isolated dystonic features particularly torticollis and spastic dysphonia*,” while infrequentely reporting CD in the detailed case descriptions. The same is true for other symptoms. Therefore we also provided in Table 1 and in the Results section the frequency of clinical signs over number of documented presence or absence of signs. True frequency of a given sign is probably intermediate between the percentages given in the two last rows of Table 1. The rarity of this disease, with just a few dozen cases reported, suggests these numbers should be viewed with caution. Also, “choreiform movements” frequently reported in the Parker series most likely reflect rapid phasic dystonic movements. This study also suffers from all classical biases inherent in a review, the biggest one being the fact that it is a systematized review, not a systematic review, without any “gray literature” search.

## Conclusion

This anthology provides a detailed review of the clinical features of DYT‐TUBB4A cases, with videos illustrating the phenotype. GPi‐DBS is a reasonable option in generalized cases, including those with the “hobby horse” dystonic gait. Botulinum toxin injections should be preferred in more focal or segmental dystonias. A trial of propranolol and/or tetrabenazine could be considered from the reported results of 1 case series. General screening for mutations in TUBB4A in isolated spasmodic dysphonia is not routinely recommended, as DYT‐TUBB4A remains an exceedingly rare cause of laryngeal dystonia. It is also a very uncommon cause of other isolated focal or segmental dystonia, as only one single large family and 3 isolated cases have been described prior to the present case series. However, certainly screening is justified in (i) individual patients combining laryngeal dystonia and dystonia in another location and (ii) in families comprising both patients with laryngeal dystonia and patients with other types of isolated dystonia. Next generation sequencing will almost certainly identify more cases of DYT‐TUBB4A, and probably expand and define the phenotype further.

## Author Roles

(1) Research project: A. Conception, B. Organization, C. Execution; (2) Statistical Analysis: A. Design, B. Execution, C. Review and Critique; (3) Manuscript: A. Writing of the first draft, B. Review and Critique.

JFB: 1B, 1C, 3A

DSK: 1C, 3A

CF: 1C, 3A

SC: 1B, 1C, 3B

FPSJr: 1C, 3B

ERB: 1C, 3B

LJO: 1A, 1B, 3B

PCA: 1A, 1B, 3B

AEL: 1A, 1B, 3B

## Disclosures

**Ethical Compliance Statement:** Brazilian families were screened as part of a research project on genetics of dystonia, approved by the institutional review board of each participating center. The Canadian and American families were screened after the probands sought medical attention at our movement disorders centers. All subjects provided written informed consent. We confirm that we have read the Journal's position on issues involved in ethical publication and affirm that this work is consistent with those guidelines.

**Funding Sources and Conflicts of Interest:** This work was supported by grants from NIH NS087997 (LJO) and FAPESP 2014/17128–2 (PCA). The manuscript has not been previously published and is not under review at any other journal. No other related work is under submission elsewhere. All authors of the paper have participated in the study, revised the manuscript and approved the final version of the manuscript. There is no ghost writer. There is no financial or any other type of conflict of interest.

**Financial Disclosures for the Previous 12 Months:** JFB: Registration fee for a neurology congress and advisory board paid by Abbvie. DSK: Drew Kern has served as an advisor for Colorado Clinical and Translational Sciences Institute (CCTSI) Data Safety Monitoring Board, and AbbVie Pharmaceutics; received honorarium from AbbVie Pharmaceutics and Boston Scientific; received grants from the National Institutes of Health, Medtronic and Boston Scientific. CF: Supported by an Edmond J. Safra Fellowship from the Michael J. Fox Foundation. SC: Honoraria from Roche and Teva Pharmaceuticals. FPSJr: Employment Hospital Israelita Albert Einstein (Sao Paulo, SP‐ Brazil). ERB: Nothing to declare. LJO: NIH grants, Patent Royalties from Athena Diagnostics, Inc. PCA: Received research grants from São Paulo research Foundation (FAPESP)2017/24022–4; Employment Hospital Israelita Albert Einstein (Sao Paulo, SP‐ Brazil). AEL: Received support for advisory work from Biogen, Janssen, Lundbeck, Merck, Roche, Sun Pharma, Theravance, and Corticobasal Degeneration Solutions; honoraria from Sun Pharma and AbbVie; grants from Brain Canada, Canadian Institutes of Health Research, Corticobasal Degeneration Solutions, Edmond J Safra Philanthropic Foundation, Michael J. Fox Foundation, the Ontario Brain Institute, Parkinson Foundation, Parkinson Society Canada, and W. Garfield Weston Foundation.
